# Mental Health Outcomes in Barcelona: The Interplay between Gentrification and Greenspace

**DOI:** 10.3390/ijerph18179314

**Published:** 2021-09-03

**Authors:** Montserrat Zayas-Costa, Helen V. S. Cole, Isabelle Anguelovski, James J. T. Connolly, Xavier Bartoll, Margarita Triguero-Mas

**Affiliations:** 1 Institute for Environmental Science and Technology, Universitat Autònoma de Barcelona, 08193 Barcelona, Spain; helen.cole@uab.cat (H.V.S.C.); isabelle.anguelovski@uab.cat (I.A.); james.connolly@ubc.ca (J.J.T.C.); margarita.triguero@uab.cat (M.T.-M.); 2 IMIM (Hospital del Mar Medical Research Institute), Barcelona Biomedical Research Park, 08003 Barcelona, Spain; 3 Barcelona Lab for Urban Environmental Justice and Sustainability, 08003 Barcelona, Spain; 4 Institució Catalana de Recerca i Estudis Avançats (ICREA), 08010 Barcelona, Spain; 5 School of Community and Regional Planning, University of British Columbia, Vancouver, BC V6T 1Z1, Canada; 6 Agència de Salut Pública de Barcelona, 08023 Barcelona, Spain; xbartoll@aspb.cat; 7 Mariana Arcaya’s Research Lab, Department of Urban Studies and Planning, Massachusetts Institute of Technology, Cambridge, MA 02138-02142, USA

**Keywords:** greenspace, green space, mental health, gentrification, parks, greenways

## Abstract

Greenspace is widely related to mental health benefits, but this relationship may vary by social group. Gentrification, as linked to processes of unequal urban development and conflict, potentially impacts health outcomes. This study explores the relationships between greenspace and mental health and between gentrification and mental health associations. It also further examines gentrification as an effect modifier in the greenspace–mental health association and SES as an effect modifier in the gentrification-mental health association. We used cross-sectional Barcelona (Spain) data from 2006, which included perceived mental health status and self-reported depression/anxiety from the Barcelona Health Survey. Greenspace exposure was measured as residential access to (1) all greenspace, (2) greenways and (3) parks in 2006. Census-tract level gentrification was measured using an index including changes in sociodemographic indicators between 1991 and 2006. Logistic regression models revealed that only greenways were associated with better mental health outcomes, with no significant relationship between mental health and parks or all greenspace. Living in gentrifying neighborhoods was protective for depression/anxiety compared to living in non-gentrifying neighborhoods. However, only residents of gentrifiable census tracts benefited from the exposure to greenways. SES was not found to be an effect modifier in the association between gentrification and mental health. Future research should tackle this study’s limitations by incorporating a direct measure of displacement in the gentrification status indicator, accounting for qualitative aspects of greenspace and user’s perceptions. Gentrification may undermine the health benefits provided by greenspace interventions.

## 1. Introduction

Two of the main challenges that cities face are the climate crisis and unequal urban development and their related social tensions, two processes that are connected and can affect health equity [1]. In recent decades, popular trends in urban policy include “health in all policies”—that is, acknowledging that all types of policies can improve and ultimately affect human health, and that cities should promote environmental improvements as health interventions [2]. For instance, to tackle the climate crisis, and implicitly improving health for urban residents, cities are incorporating and reinforcing green interventions such as greenspaces (parks, gardens, greenways, etc.) [3], which have been proven to provide a wide range of cooling, recreational or flood-proofing benefits for residents. However, those green interventions may in practice reinforce uneven urban development patterns and inequality among different social groups [4] (i.e., privileged residents, such as white affluent communities, generally have better access to environmental amenities [5,6]). At the same time, more vulnerable neighborhoods, such as those predominantly inhabited by racialized minorities and low-income residents, are left at risk for gentrification, an unequal process of urban redevelopment which tends to benefit these same privileged wealthy residents [7,8]. Thus, the way that access to greenspace is distributed among social groups may play a significant role in health equity in cities.

### 1.1. Are Greenspaces Always Associated with Health Benefits for Urban Residents?

Exposure to greenspace has been widely associated with multiple beneficial health outcomes, such as lower mortality [9], better self-reported health and better mental health outcomes in urban populations, including general mental health, psychological well-being, perceived mental health, stress, depression, anxiety and mood disorder treatment [10,11,12]. Mechanisms that have been theorized to potentially explain these relationships include: increasing physical activity, enhancing social contacts, restoring capacities—by reducing stress and restoring attention—and decreasing exposure to environmental hazards [13,14]. The type and quality of greenspace may be determinants in producing health benefits, as they may promote variable psychological responses [15]. For instance, different asthma and obesity outcomes have been found for children depending on their exposure to parks or forests [16]. However, little research has been conducted regarding types of greenspaces (for exceptions see [15,16]).

Some evidence has shown that the health effects of greenspace exposure may vary by sociodemographics, such as age [17,18], gender [12,19], care provider status—which might increase greenspace exposure—ethnicity/race [20] or socioeconomic status (SES) [20,21,22]. Indeed, several studies have demonstrated that the health benefits of greenspace may be modified by SES, being stronger for those with low SES [18,21,23]. However, another factor which may modify the association between greenspace and health is gentrification [7] and the gentrification status of one’s neighborhood [22].

### 1.2. Are Greenspaces Associated with Neighborhood Gentrification Processes?

Gentrification is a process that occurs when historically disinvested neighborhoods experience economic revitalization that transforms their demographic, real estate and business characteristics [24], leading to an influx of new residents of higher SES and social privilege, and a transition towards a more educated, wealthier, whiter population, able to afford new or renovated, more expensive properties while also fomenting new cultural and consumption practices, finally changing the essential character of the neighborhood [22,25,26,27]. The association between new neighborhood resources, such as the creation or restoration of greenspaces, and gentrification has been shown in a variety of cities worldwide [28], including Barcelona [29,30,31,32]. However, the direction of the causal pathway remains unclear [28,33]. It could either be that greenspaces catalyze gentrification by providing different environmental amenities that strengthen the identity of an area as an attractive and desirable place to work, live and visit, with upward effects on local economies and real estate values [34]; or, instead, that the resources applied to the creation of the amenities are a result of gentrification processes that bring residents with greater power and influence, which may be used to advocate for new greenspaces for instance, into previously distressed neighborhoods [7].

### 1.3. Is Gentrification Associated with the Health of Urban Residents?

Emerging research on the potential effects of gentrification on health has produced contradictory findings. Gentrification, at a neighborhood level, has positive economic impacts in impoverished districts due to the reintroduction of resources by middle- and upper-class households [25,27]. For example, newly affluent neighborhoods may experience an increase in business and employment opportunities [25,35], new commerce, better quality food options, increased property values for homeowners, etc. At the same time, wealthier residents may introduce financial resources to these neighborhoods, which may influence the resources a city spends on renewing or regenerating the area, leading to physical improvements such as cleaner streets and improved access to amenities and city services [36].

Overall, those socioeconomic improvements are associated with increased quality of life and in turn better self-rated health, at a neighborhood-level [27,36,37]. However, concerns arise as to how well these benefits may be distributed among social groups, including marginalized populations who are being gentrified [38]. For example, the new cultural and consumption patterns brought about by gentrification have been shown to disrupt the traditional cultural and social fabric of a neighborhood. Moreover, the influx of new residents usually leads to “whitening” neighborhoods, and thus, to an increased exposure to racism [36]. With public spaces, long-term families can see their use of greenspaces diminished due to the high presence of newcomers, newcomers’ use of greenspaces and the new norms they establish [24,39]. In sum, all these processes contribute to potential cultural displacement [40,41].

In addition to cultural displacement, increased cost of living through both mostly housing and food prices in gentrifying neighborhoods can in turn result in a potential physical displacement, as marginalized residents are priced out [40]. In more extreme cases, as a process of planned gentrification, banks and investment funds engage in “property mobbing”, that is harassment of residents to evict them before rehabbing buildings for wealthy foreigners [24]. Property mobbing can lead to mental health disorders such as chronic stress, depression or anxiety and suicidal thought patterns, as a qualitative study has shown [24]. Indeed, a study concluded that living in gentrified neighborhoods was associated with increased likelihood of serious psychological distress relative to living in a low-income and not gentrified neighborhood: gentrification appeared to have a negative impact on the mental health of renters, low-income residents and long-term residents. This effect was not observed among higher-income residents and recent residents [42].

### 1.4. Gentrification as an Effect Modifier of the Association between Greenspaces and Health

While the effect modification of SES in the greenspace–health association has been studied on various occasions [9,17,18,21,43], the potential role of gentrification as an effect modifier has been rarely explored. Redeveloping, gentrifying urban neighborhoods are, indeed, exposed to complex environmental riskscapes, which are shown to create new patterns of urban health inequality [44]. For example, one recent study in several cities in North America and Europe found that when gentrification processes and improvements to greenspaces co-existed in the same neighborhood, underprivileged neighborhood residents were perceived to experience new or improved greenspaces as what we call “disruptive green landscapes”, i.e., spaces with which they were not physically or emotionally engaged and that were not benefiting their health [39]. Moreover, one study has quantitatively explored the potential role of gentrification as an effect modifier of the association between greenspace and health, in the North American context, finding that only people living in gentrifying neighborhoods reported health benefits from greenspaces [22]. Furthermore, this study focused only on perceived general health despite that both greenspace exposure and gentrification have implications for mental health. In sum, despite these emerging studies, more expanded research needs to disentangle the complex relationship between green space, health and gentrification.

### 1.5. Specific Aims

This study aims to understand the interrelations between greenspaces, health and gentrification in the European context by: (1) exploring the associations between greenspace (overall and different typologies separately) and mental health, (2) investigating the relationships between gentrification and mental health, (3) if the previous associations exist, assessing whether gentrification plays a role in modifying the association between greenspace exposure and mental health, on the one hand, and (4) examining if SES interacts in the association between gentrification and mental health, on the other hand.

## 2. Materials and Methods

### 2.1. Study Design and Population

This study is a cross-sectional study using data from 2006, to avoid any possible confounding effect by the Spanish financial crisis starting in 2008, which deeply disturbed housing and redevelopment markets and created intense waves of evictions. Initially, the 2006 Barcelona Health Survey (BHS) did a probabilistic stratified non-proportional sample representative of the adult population of the city of Barcelona. The 2006 BHS sample included 6108 adults. From that sample, we included in our analyses those individuals with geocoded residences (n = 2513) and for whom perceived mental health or depression/anxiety data were not missing (*n* = 2425; *n* = 2450, respectively). The sample included in this study did not show any significant differences when compared to the 2006 BHS sample.

### 2.2. Data Sources

#### 2.2.1. Health Data

The study employed two measures of mental health outcomes that were included in the BHS. First, general mental health was measured by the Goldberg “General Health Questionnaire” (GHQ-12), a 12-item scale that is a valid mental health screening instrument for detecting both the inability to continue with normal activities and new appearances of distress [45]. Each item is responded to using a four-point Likert scale and a final score was calculated by summing the responses from the 12 items. The scores obtained were then categorized into a dichotomous variable, where values of ≥ 3 were classified as poor mental health [45] (Appendix A). Second, self-reported depression and/or anxiety— not necessarily diagnosed—was measured as a dichotomous (yes/no) answer to the question “Do you have or have suffered from depression and/or anxiety?” comprised in the BHS.

#### 2.2.2. Greenspace Exposure Data

Greenspace exposure was characterized with ArcGIS using network buffers of 300 m around the geocoded address of residence of each BHS participant. We used 300 m network buffers because this is what is universally considered a generic walkable distance [12]. By manually verifying all greenspace that existed in 2006 using a local database used for managing greenspaces in the city, greenspaces were categorized by type of use: greenways, gardens, parks, recreation spaces and preserve areas. Then, the variables representing the percentage of area covered with each type of greenspace were categorized into dichotomous variables using the 75th percentile as a threshold, indicating “high presence” (% > p75) or “low or no presence” (% ≤ p75) of greenspace. In this study, three variables were used, indicating presence of greenways, parks and a global variable of all greenspace with a 300 m network buffer.

#### 2.2.3. Gentrification

We calculated a gentrification score from data obtained from the Catalan Statistics Institute and the Department of Statistics for the years 1991 and 2006 that reflected the changes in sociodemographics (percentage of ethnically marginalized, percentage of residents with university degree or higher, percentage of residents with high-income) and real estate prices for this period of time at the census tract level. Census tracts were categorized as: 1) gentrifiable (i.e., census tracts which had a mean income at or under the city-wide mean for Barcelona in 1991—considered non-predominantly wealthy—with low gentrification scores—under the threshold set at the 90th percentile), 2) gentrifying (i.e., non-predominantly wealthy tracts experiencing gentrification on the 90th percentile or above), (3) super-gentrifying (i.e., wealthy tracts in 1991 (above the mean income) that still had some room to experience more intense gentrification by 2006 (score at or above the 90th percentile)) or (4) wealthy (i.e., census tracts which had a mean income above the city-wide mean and with gentrification scores under the 90th percentile) (Appendix B).

#### 2.2.4. Covariates

Other variables were sex, age, ethnicity—that is, being of a marginalized ethnic group assessed as having nationality from the Global South (African countries, Philippines, Peru, Pakistan, Bolivia, Ecuador, Colombia or Dominican Republic), as used in other studies [8]—and caregiver status—that is, being a care provider to children ≤ 14 and/or elderly ≥ 65 and/or disabled people, which we hypothesize increases greenspace exposure. Individual socioeconomic data were assessed according to the Spanish Epidemiology Society [46]. The BHS question about previous or current occupation was coded following the 1994 National Occupation Classification and then grouped into six categories (I, II, III, IVa, IVb, V), based on the British Registrar General Classification [45]. The six categories were then recategorized into two groups: manual (I, II, III) and non-manual (IVa, IVb, V) occupations [45].

### 2.3. Statistical Analysis

Univariate and bivariate descriptive analysis were conducted for all variables. Then, weighted logistic regression models were performed to explore the relationship between greenspace or gentrification as the exposure variables and the two mental health outcomes. Each of these models were adjusted to scale the data to the 2006 Barcelona population by utilizing an individual weight variable provided with the dataset (BHS-2006) and the *svyset* STATA command. In addition, the models included the following covariates: sex, age, ethnicity, SES, caregiver status and neighborhood as a control variable to approximate spatial clustering at a district level [22]. Models for each of the greenspace variables and for gentrification status were developed, testing one exposure variable at a time. Interactions were tested for gentrification and for SES, testing the null hypothesis of homogeneity and conducting a Wald test. We used STATA-SE 64, version 15.0 and set statistical significance at P < 0.05.

Sensitivity analyses were conducted to evaluate the robustness of our findings. The “caregiver status” was suppressed from the models to explore whether this variable—not previously studied in the literature as a whole despite that only having children has been included as a confounder in previous studies [47]—could be interfering with our findings. We repeated the aforementioned main analyses taking the exposure variables in 100 m and 500 m network buffers and 300 m circular buffers to evaluate the consistence of our selection of 300 m network buffers.

## 3. Results

### 3.1. Study Characteristics

The characteristics of study participants, prevalence of the outcomes and description of indicators of greenspace and gentrification are presented in Table 1, weight adjusted. There were 2425 (96.50%) valid responses for the perceived mental health outcome and 2512 (99.96%) valid responses for the depression and/or anxiety health outcome. For analysis involving SES (i.e., regression models), the sample size resulted in 2367 when analyzing mental health and 2450 when analyzing depression/anxiety, as there were 62 individuals missing data for SES. Our sample had a low percentage of ethnically marginalized participants (around 8%), almost half of the sample had a low SES (48%) and approximately a quarter (around 24%) of them were caregivers. Around half of the sample lived in gentrifiable areas (54%), almost 35% in wealthy neighborhoods and the rest in gentrifying (under 8%) and super-gentrifying (nearly 4%) census tracts.

Overall, 16.03% of respondents experienced poor perceived mental health (n = 376) and 16.02% reported depression and/or anxiety (n = 389). Some greenspace typologies appeared to have a very low presence: only <7% of residents presented “high presence” of gardens, recreation spaces and preserve areas near their homes, and none of these—with the exception of preserve areas vs depression/anxiety—presented statistically significant associations with mental health outcomes. Regarding the greenspace typologies showing a high presence (>7%) among the sample, no significant differences in all greenspace nor parks were found by perceived mental health status nor depression/anxiety, but people with a high presence of greenways around their residences presented a marginally or statistically significant lower prevalence of a poor perceived mental health (P = 0.066) and of having had depression/anxiety (P = 0.027), respectively. However, there were several differences in mental health status and in presenting depression and/or anxiety between sociodemographic population groups: women, manual workers, marginalized ethnic groups and caregivers had statistically significantly higher prevalence of worse perceived mental health. Regarding depression/anxiety, women and manual workers also presented a statistically significant higher risk of having depression/anxiety; moreover, those with depression/anxiety had a statistically significant higher mean age than those without depression/anxiety. Statistically significant differences were shown in having depression and/or anxiety by gentrification status (P = 0.016): gentrifiable census tracts showed the biggest differences in presenting depression/anxiety (62%) or not (55%), followed by wealthy census tracts (31% vs 34%), gentrifying census tracts (4% vs 8%) and supergentrifying census tracts (3% vs 4%).

### 3.2. Model Results

Among all greenspace typologies, only three variables were modeled in this study: all greenspace, greenways and parks. The other greenspace variables (gardens, recreation spaces and preserve areas) presented a very homogeneous distribution among the sample, as most of our participants were very lowly exposed to these types of greenspaces (Barcelona as a city has few of those spaces in comparison with other cities) (Table 1); therefore, they were excluded from our models.

#### 3.2.1. Are Greenspaces Associated with the Health of Urban Residents?

When looking at the greenspace exposure models, adjusting for sociodemographics, the odds of having poor mental health and of having depression/anxiety did not vary by exposure to all greenspace or to parks (Table 2). However, those living near a high presence of greenways had a statistically significant 30% lower likelihood of reporting poor perceived mental health and a significant 34% lower likelihood of having depression/anxiety.

#### 3.2.2. Are Greenspaces Associated with Neighborhood Gentrification Processes?

On the other hand, gentrification status as the exposure variable in the adjusted models did not happen to be statistically significantly associated with perceived mental health. However, those living in gentrifying census tracts showed a statistically significant 57% lower odds of presenting depression/anxiety (P = 0.003) than those living in gentrifiable census tracts, but no differences in having depression/anxiety appeared for wealthy or supergentrifying census tracts when compared with gentrifiable census tracts.

In all adjusted models (both the ones exploring greenspace and those exploring gentrification status as the exposure variable), the associations observed in the bivariate analysis between perceived mental health and sociodemographic variables were maintained for women, ethnic minorities and caregivers. Additionally, manual worker classes showed a marginally statistically significant association with poor mental health in the parks and the all greenspace models and, in the greenways model, manual classes presented a significant 29% higher odds of having poor mental health than non-manual classes.

The results found in the bivariate analysis between depression/anxiety and sociodemographic variables were also maintained in the greenspace and the gentrification status adjusted models: women, older people and manual workers showed higher odds of presenting depression/anxiety.

#### 3.2.3. Gentrification as an Effect Modifier of the Association between Greenspaces and Health

On the one hand, greenways appeared to be significantly associated to lower odds of depression/anxiety; on the other hand, gentrification status was also significantly linked to reported depression/anxiety. Therefore, interaction terms were assessed for gentrification status in the association between greenways exposure and reported depression/anxiety. We found that there exists a statistically significant effect modification of gentrifying census tracts in the association between greenways and depression/anxiety (Table 3). Stratified models by gentrification status revealed that the significant relationship between exposure to greenways and lower likelihood of reporting depression/anxiety remained only for residents of gentrifiable neighborhoods (OR = 0.61, 95% CI (0.41, 0.90)), but not for gentrifying, wealthy nor supergentrifying neighborhoods (Table 4).

#### 3.2.4. SES as an Effect Modifier of the Association between Gentrification and Health

Interaction terms were assessed for SES in the association between gentrification status exposure and reported depression/anxiety, finding null results in SES as an effect modifier.

### 3.3. Sensitivity Analysis

In sensitivity analyses, the exclusion of the “caregiver status” variable in the models did not provide any different results for the main analysis (Table A2, Table A3. Greenspace variables at 100 m network buffer and 300 m circular buffer did not show any significant associations with any of the health outcomes; 500 m network buffer only presented a marginal protective effect for greenways for both mental health outcomes (Table A2).

## 4. Discussion

Our results showed that, in our sample, participants having a high presence of greenways with a 300 m network buffer around their residence had lower odds of poor perceived mental health and reported depression/anxiety than those having a low or no presence of greenways with a 300 m network buffer around their residence. Moreover, we found that, overall, participants living in gentrifying census tracts had lower odds of having had depression/anxiety than those living in gentrifiable neighborhoods. However, only people living in gentrifiable neighborhoods benefited from the exposure to greenways in reporting depression/anxiety, which has important implications for green space and health equity.

### 4.1. Greenspace—Mental Health

Our findings of high residential exposure to greenways being associated or marginally associated with lower risk of poor mental health (both self-perceived poor mental health and having had depression/anxiety) but no associations for other greenspace typologies had already been described in the literature [48]. A study in Berlin that examined the effect of greenways and parks on residents concluded that greenways provided more benefits than parks; this difference was explained by the ways in which various types of greenspaces are used by residents.

In that study, participants who regularly used a vegetated trail along a canal—at least once a week—had significantly lower cortisol levels and reported significantly higher life satisfaction than less frequent users. In addition, qualitative analysis of users’ specific uses and perceptions of the trails supported the hypothesis that frequent users of this space had frequent chances for restoration; some participants appreciated the opportunity for “escaping from routines”, “quickly stretching one’s legs” or “breathing fresh air”. It also showed that the trails were predominantly used for recreation in leisure time, such as promenading and social activities, but also for daily commutes, even if they were detours, for routes by foot or bicycle and for running. Moreover, some expressed their high satisfaction with their neighborhood due to this trail. Additionally, the benefits were higher than those found for parks [48].

Greenways’ connectivity and easier accessibility could explain that simply walking on a greenway on the way to work or school may produce positive effects on mental health [49]. This is aligned with previous findings, which highlighted the importance of surrounding greenness, such as street trees, paths and greenways, for providing more effective opportunities for restoration/stress reduction through, for example, visual access, than other types of greenspaces [12]. At the same time, residential proximity to greenways has been linked to increased frequency of walking and practicing moderate physical activity [49]. They may also be appealing by simultaneously offering good connectivity and allowing the practice of physical activity for recreational purposes (e.g., biking, skateboarding or rollerblading). Barcelona in particular has a long history and tradition of strolling and social activities on greenways, the traditional well-known “Ramblas”, and those are scattered through the entire city, including in more working-class, gentrifiable neighborhoods in the historically working-class districts of Nou Barris or Sant Andreu.

The null results found for parks and for the overall greenspace variable on mental health (which includes greenways, parks, gardens, recreation spaces and preserve areas) contradict some of the existing literature that shows beneficial associations between residential greenspace exposure and mental health using a wide range of measures [10,11,12,18,19,23], but are aligned with some that did not show any significant associations [43,50,51,52]. A possible explanation for these differential results may be their limited accessibility and usability, in contrast to greenways. First, it is unclear whether parks in our measurement are always accessible to the general public. Two of the main, largest park spaces in Barcelona—Montjuïc and Collserola—are difficult to access and some of the more central ones—Parc de la Ciutadella—tend to be overcrowded with tourists. For instance, a study showed that easy access to a place to exercise results in a 5.1 percent median increase in aerobic capacity, along with weight loss, a reduction in body fat, improvements in flexibility and an increase in perceived energy, which is linked to mental health [53]. Second, some of the pathways linking greenspace to mental health depend on active use, which may depend on a series of underlying behavioral factors. Many of the studies linking parks with better mental health are based on individuals who have purposefully chosen to exercise or spend recreation time in parks [53]. Third, safety or quality concerns may play a role in the greenspace usability. For example, drug-dealing or drug use in local parks may debilitate structures of informal social care and limit opportunities to use public space [24]. Additionally, quality characteristics of greenspaces, such as the presence of sports amenities in recreation areas, may interfere in the physical activity or the social cohesion pathways in ameliorating mental health [54]. However, the present study was unable to determine the type of use of these greenspace typologies by the respondents near their homes.

Another possible explanation could be that sociodemographic determinants of presenting poor mental health—for instance, that women, those with lower SES, ethnic minorities and caregivers are more likely than others to report poor mental health, both perceived mental health and depression/anxiety—may prevent the visibility of the effect of greenspace if such sociodemographic differences account for a large amount of the difference in outcomes. In fact, in our models, gender, age and in some ethnicity and SES, emerged as strongly significantly related with the mental health outcomes.

Regarding the greenspace variables buffer election, the buffer size was elected following the WHO recommendations, which state that 300 m linear buffer is an adequate measurement of greenspace, as they indicate that “network distance may be more accurate and reflect variation in local access routes” [55,56].

### 4.2. Gentrification—Mental Health

Findings from the current study indicate residents of census tracts experiencing gentrification reported having lower odds of having depression/anxiety than residents living in gentrifiable census tracts. The protective effect of gentrifying neighborhoods has also already been found in previous literature: a study found that gentrification was significantly and positively related with self-rated physical neighborhood health outcomes, at a city scale [27]. Other studies have found a potential for gentrification to improve resident health for the population at large [36,57,58], but in some studies, worse health outcomes were found for marginalized residents living in such neighborhoods. For instance, living in a gentrifying neighborhood was associated with worse self-rated health among Black residents [36] and worse birth outcomes for Hispanic and Black women [57,58,59].

As already described, the positive socioeconomic impacts that derive from gentrification may bring a greater availability of resources that could be associated with improvements to quality of life and in turn better self-rated health overall [36], explaining why residents of gentrifying neighborhoods show a significant protective effect against depression/anxiety compared to residents of gentrifiable areas.

On the other hand, no changes were found for perceived mental health by gentrification status, as has been reported in other studies [37,59]. The null effect of gentrification in perceived mental health, in contrast with the significant results found for depression/anxiety, could be due to an outcome misclassification; the question of “having had depression/anxiety” might be answered in a more direct way as perceived as a more extreme outcome than the 12-item questionnaire of perceived mental health.

### 4.3. Effect Modification of Gentrification

While gentrification favors mental health overall, a differential effect was seen with how residents benefit from greenways by gentrification status. Only residents of gentrifiable census tracts experienced a lower odds of reporting depression/anxiety, but no benefits were found among residents of gentrifying, wealthy nor supergentrifying census tracts. This is aligned with our hypothesis that gentrification may undermine the beneficial effects of greenspace interventions for mental health. One recent study [39] argued that, in gentrified neighborhoods, greenspaces become disruptive green landscapes: gentrification has found to be linked to feeling unwelcome and socially controlled in natural outdoor environments and, also, to conflicts between users. They often emerge as contested spaces in neighborhoods undergoing gentrification processes. In Barcelona, greenways are at times overtaken spaces by tourists, especially those in gentrifying neighborhoods, which might explain their lack of appeal and use among locals—a widely reported and known trend in the city and even the object of documentaries (https://smoda.elpais.com/placeres/bye-bye-barcelona-el-documental-contra-el-turismo-masivo/, accessed on: 11 August 2021). All in all, gentrification may trigger socio-spatial inequalities, privilege, exclusion and socio-cultural, economic and physical displacement from greenspaces, which may prevent residents of gentrified neighborhoods to experience mental wellbeing, contrarily to non-gentrified neighborhoods, which experience mental health benefits from greenways [39].

However, these health benefits were expected to be differential among groups, showing poorer health outcomes for more underprivileged groups, as some studies have shown [22,27,39,60]. Our results do not corroborate these, as we saw no differential effect of gentrification on mental health by SES group. This phenomenon could be explained by some of the study limitations.

First, it is possible that there exists a delay related to the effect of gentrification on health that the present study could not capture. Some of the literature shows evidence for long-term residents of gentrifying neighborhoods experiencing a profound change and alienation, the breakdown of informal place-based networks of exchange, the loss of gathering spaces and institutions, symbolic manifestations and socioeconomic inequality. However, these effects may not impact working-class residents who had recently moved to the neighborhood [37]. Additionally, there has not been time enough for physical displacement to be that widespread, at least in the earlier stages of gentrification [37]; therefore, the effects of gentrification on the population may take some time to emerge.

Second, it might be that the neighborhoods were so gentrified already in 2006 that those remaining in gentrifying neighborhoods were mostly the ones who had more resources to stay—i.e., homeowners—or those who had a greater income, as physical displacement had already occurred. A more accurate description of the groups’ compositions, which would help better identify displacement threats, would help enlighten this mechanism. It is worth pointing out that the study is temporarily placed on a historic impasse on Barcelona’s greening and urbanism evolution: 2006 is substantially after the major green effort that occurred to prepare for the Olympic games, but also before the uncontrolled wave of tourism—which is tightly linked to gentrification in Barcelona. Therefore, the effects of these events on health are difficult to place on a specific time-lapse; it may be worth repeating the study with much more recent data, when the full impact of the current greening efforts and new patterns of tourism and potential gentrification have already played out.

### 4.4. Strengths and Limitations

Our study has several strengths. Few studies have investigated the greenspace–health associations in a southern European population [12]. Additionally, in this manuscript, we explore different greenspace typologies and the links of mental health and gentrification, which have not been widely previously studied [7,36]. Our health, gentrification and greenspace exposure data were collected using validated and/or objective methods. Greenspace was exhaustively measured with high quality quantitative data manually checked using satellite images and assessed using network buffers, producing a robust measure. Our probabilistic sampling strategy and the inclusion of a sampling weight allowed us to produce representative estimates of the explored associations for the whole Barcelona population in 2006.

This study also has some limitations, such as the inherent cross-sectional design limitations, and thus, causality cannot be tested. Using self-reported questionnaires could result in outcome misclassification and social desirability biases. Additionally, a larger sample size, especially knowing the overall population and extension of Barcelona, would ensure stronger statistical power. Accounting for the time spent in the residential area or frequency of visits to greenspace would be more reliable. Qualitative data would better capture additional factors that influence the extent to which people engage with their local greenspace [43], such as programming of greenspace or user’s perceptions [10], and it may help enlighten some less well explored pathways, such as viewing greenspaces from windows [61] and childhood experiences with nature [18]. Although the buffer covered areas next to bluespace, including direct measures of bluespaces, would be important as they may also have a positive effect on health [11]. Our gentrification measure did not consider length of residence, displacement, nor a direct measure to distinguish the whole profile of “gentrifiers” from prior residents. Additionally, even if the gentrification measure and unit of scale (census tract) is based on previous studies, alternative measures may yield different results [36,62].

## 5. Conclusions

The literature has reported greenspace to be strongly related to mental health benefits. Simultaneously, gentrification is also thought to affect resident’s health. In this study, we assessed the interplay between greenspaces and gentrification status on mental health. This research presented partially statistically significant results. Greenways were associated to better mental health outcomes, signaling the importance of incorporating these in urban planning. However, parks and the overall greenspace variable did not show statistically significant associations with mental health, probably due to their more limited access and usability in the case of Barcelona. On the other hand, gentrification was associated with mental health benefits among residents overall, but further examination revealed that this benefit only held for residents of gentrifiable areas, areas that had basically not been gentrified yet. These findings corroborate that gentrification may undermine health benefits provided by green interventions, possibly due to a disruptive effect of green landscapes and, thus, health justice goals. The findings drive some reflections: future research should incorporate qualitative aspects of greenspace and user’s perceptions and effective use of those spaces. When these aspects are acknowledged, mechanisms why some populations differently benefit from greenspaces would be better captured, which is useful for urban planning to program equitable green interventions.

## Figures and Tables

**Table 1:** Characteristics of study participants for all sociodemographic, gentrification status and greenspace variables by perceived mental health status and depression/anxiety (weight-adjusted).

	Mental Health						Depression or Anxiety				
	*n*	Total ^a^	Good Perceived Mental Health (*n* = 2049, 83.97%)	Poor Perceived Mental Health (*n* = 376, 16.03%)	P-Value		*n*	Total ^a^	Not Having Depression/Anxiety (*n* = 2123, 83.98%)	Having Depression/Anxiety (*n* = 389, 16.02%)	P-Value
**Sociodemographics**											
Gender, females	2425	1289 (53.24%)	1027 (49.98%)	262 (70.33%)	<0.0001		2512	1339 (53.34%)	1071 (50.42%)	268 (68.64%)	<0.0001
Age [years: mean (sd)]	2425	49.05 (18.80)	48.94 (18.76)	49.67 (19.07)	0.486		2512	49.68 (19.28)	48.57 (19.16)	55.73 (18.86)	<0.0001
Ethnicity, being of a marginalized ethnic group	2425	139 (8.05%)	110 (7.28%)	29 (12.07%)	0.014		2512	143 (7.95%)	122 (7.89%)	21 (8.25%)	0.851
Socioeconomic status, manual	2367	1124 (48.11%)	922 (46.86%)	202 (54.66%)	0.010		2450	1177 (48.63%)	950 (46.51%)	227 (59.80%)	<0.0001
Being a caregiver, yes	2425	562 (23.76%)	446 (22.36%)	116 (31.12%)	0.001		2512	562 (22.95%)	479 (23.00%)	83 (22.69%)	0.900
**Gentrification status**	2425				0.901		2512				0.016
Gentrifiable		1306 (53.86%)	1091 (55.02%)	215 (57.09%)				1368 (55.98%)	1128 (54.76%)	240 (62.37%)	
Gentrifying		197 (8.12%)	168 (7.12%)	29 (6.90%)				202 (6.98%)	183 (7.55%)	19 (3.98%)	
Wealthy		836 (34.47%)	719 (34.29%)	117 (32.32%)				851 (33.42%)	734 (33.97%)	117 (30.53%)	
Supergentrifying		86 (3.55%)	71 (3.57%)	15 (3.67%)				91 (3.62%)	78 (3.72%)	13 (3.12%)	
**Greenspace exposure: > p75 Percentage of 300 m network buffer covered with**											
All greenspace	2425	605 (24.95%)	514 (25.24%)	91 (24.24%)	0.699		2512	627 (24.96%)	532 (25.24%)	95 (23.97%)	0.609
Greenways	2425	607 (25.03%)	529 (27.88%)	78 (22.73%)	0.066		2512	627 (24.96%)	550 (27.89%)	77 (21.89%)	0.027
Parks	2425	608 (25.07%)	507 (24.26%)	101 (25.73%)	0.558		2512	629 (25.04%)	530 (24.45%)	99 (24.10%)	0.885
Gardens	2425	23 (0.95%)	20 (0.94%)	3 (0.55%)	0.412		2512	24 (0.96%)	21 (0.96%)	3 (0.59%)	0.466
Recreation spaces	2425	152 (6.27%)	126 (6.82%)	26 (7.58%)	0.628		2512	158 (6.29%)	131 (6.91%)	27 (7.56%)	0.675
Preserve areas	2425	36 (1.48%)	29 (1.35%)	7 (1.81%)	0.489		2512	36 (1.43%)	24 (1.06%)	12 (3.03%)	0.002

^a^ Absolute and relative frequencies are shown for all categorical variables [*n* (%)]. For continuous variables, mean (standard deviation (sd)) is shown for those following normal distribution.

**Table 2:** Logistic regression models (weight-adjusted and spatial clustering) adjusted for sociodemographic variables (sex, age, ethnicity, socioeconomic status and caregiver status) (*n* = 2367 in models involving mental health; *n* = 2450 in models involving depression/anxiety).

	OR	(95% CI)	P-Value		OR	(95% CI)	P-Value		OR	(95% CI)	P-Value			OR	(95% CI)	P-Value
	Greenspace Models												Gentrification Models			
	*All greenspace*				*Greenways*				*Parks*							
Sex (ref = male)	2.33	(1.80, 3.01)	<0.0001		2.37	(1.84, 3.07)	<0.0001		2.32	(1.80, 3.01)	<0.0001		Sex (ref = male)	2.33	(1.80, 3.01)	<0.001
Age	1.00	(0.99, 1.01)	0.869		1.00	(0.99, 1.01)	0.807		1.00	(0.99, 1.01)	0.872		Age	1.00	(0.99, 1.01)	0.876
Ethnicity (ref = non-marginalized)	1.68	(1.00, 1.65)	0.027		1.70	(1.07, 2.69)	0.024		1.69	(1.07, 2.67)	0.026		Ethnicity (ref = non-marginalized)	1.69	(1.07, 2.68)	0.025
SES (ref = non-manual)	1.28	(1.00, 1.65)	0.052		1.29	(1.00, 1.66)	0.049		1.27	(0.99, 1.64)	0.059		SES (ref = non-manual)	1.27	(0.98, 1.64)	0.071
Caregiver status (ref = not being a caregiver)	1.42	(1.09, 1.85)	0.009		1.42	(1.09, 1.85)	0.009		1.42	(1.09, 1.85)	0.009		Caregiver status (ref = not being a caregiver)	1.42	(1.09, 1.85)	0.009
Greenspace variable at a 300 m network buffer	0.91	(0.69, 1.20)	0.499		0.70	(0.52, 0.93)	0.016		1.06	(0.81, 1.39)	0.664		Gentrification (gentrifiable not gentrifying)			
													Gentrifying	0.87	(0.54, 1.41)	0.577
													Wealthy	0.95	(0.72, 1.26)	0.733
													Supergentrifying	1.05	(0.57, 1.93)	0.875
Sex (ref = male)	2.09	(1.63, 2.68)	<0.0001		2.12	(1.66, 2.72)	<0.0001		2.09	(1.63, 2.68)	<0.0001		Sex (ref = male)	2.12	(1.65, 2.73)	<0.001
Age	1.02	(1.01, 1.02)	<0.0001		1.02	(1.01, 1.02)	<0.0001		1.02	(1.01, 1.02)	<0.0001		Age	1.02	(1.01, 1.02)	<0.001
Ethnicity (ref = non-marginalized)	1.23	(0.71, 2.14)	0.451		1.25	(0.72, 2.17)	0.434		1.23	(0.71, 2.14)	0.454		Ethnicity (ref = non-marginalized)	1.26	(0.73, 2.20)	0.405
SES (ref = non-manual)	1.50	(1.16, 1.94)	0.002		1.51	(1.18, 1.95)	0.002		1.49	(1.16, 1.93)	0.002		SES (ref = non-manual)	1.43	(1.10, 1.86)	0.007
Caregiver status (ref = not being a caregiver)	1.00	(0.75, 1.32)	0.978		1.00	(0.75, 1.32)	0.977		1.00	(0.75, 1.32)	0.973		Caregiver status (ref = not being a caregiver)	1.02	(0.77, 1.35)	0.913
Greenspace variable at a 300 m network buffer	0.92	(0.70, 1.20)	0.534		0.67	(0.50, 0.91)	0.009		0.96	(0.73, 1.25)	0.743		Gentrification (gentrifiable not gentrifying)			
													Gentrifying	0.43	(0.25, 0.75)	0.003
													Wealthy	0.80	(0.61, 1.05)	0.112
													Supergentrifying	0.75	(0.38, 1.49)	0.410

**Table 3:** Logistic regression models (weight-adjusted and spatial clustering) adjusted for sociodemographic variables (sex, age, ethnicity, socioeconomic status and caregiver status) with potential interactions between greenways X gentrification and SES X gentrification.

	OR	(95% CI)	P-Value			OR	(95% CI)	P-Value
		Greenways				Gentrification Models		
**Having had depression/anxiety**								
Sex (ref = male)	2.14	(1.67, 2.75)	<0.0001		Sex (ref = male)	2.12	(1.65, 2.73)	<0.0001
Age	1.02	(1.01, 1.02)	<0.0001		Age	1.02	(1.01, 1.02)	<0.0001
Ethnicity (ref = non-marginalized)	1.27	(0.73, 2.21)	0.403		Ethnicity (ref = non-marginalized)	1.27	(0.73, 2.20)	0.402
SES (ref = non-manual)	1.46	(1.12, 1.90)	0.005		SES (ref = non-manual)	1.42	(1.03, 1.96)	0.031
Caregiver status (ref = not being a caregiver)	1.01	(0.76, 1.34)	0.943		Caregiver status (ref = not being a caregiver)	1.01	(0.76, 1.34)	0.924
Presence of greenways (> p75 % 300 m network buffer)	0.62	(0.41, 0.94)	0.024					
Gentrification (ref = gentrifiable)					Gentrification (ref = gentrifiable)			
Gentrifying	0.26	(0.11, 0.57)	0.001		Gentrifying	0.26	(0.09, 0.79)	0.018
Wealthy	0.77	(0.57, 1.05)	0.104		Wealthy	0.83	(0.56, 1.22)	0.345
Supergentrifying	0.92	(0.44, 1.91)	0.823		Supergentrifying	0.64	(0.26, 1.56)	0.327
Interaction greenways X gentrification (ref = gentrifiable)					Interaction SES X gentrification (ref = gentrifiable)			
Interaction greenways X Gentrifying	3.31	(1.05, 10.42)	0.041		Interaction SES X Gentrifying	2.01	(0.55, 7.35)	0.289
Interaction greenways X Wealthy	1.31	(0.67, 2.55)	0.426		Interaction SES X Wealthy	0.91	(0.52. 1.58)	0.735
Interaction greenways X Supergentrifying	0.36	(0.04, 3.17)	0.357		Interaction SES X Supergentrifying	1.46	(0.36, 5.90)	0.596

**Table 4:** Logistic regression models (weight-adjusted and spatial clustering) stratified by neighborhood type reporting the relationship between exposure to greenways and the odds of experiencing anxiety/depression, adjusted for sociodemographic variables (sex, age, ethnicity, socioeconomic status and caregiver status).

	Gentrifiable	Gentrifying	Wealthy	Supergentrifying
	OR (95% CI)	OR (95% CI)	OR (95% CI)	OR (95% CI)
**Greenway exposure (ref = low or no presence)**	0.61 (0.41, 0.90) *	1.34 (0.46, 3.89)	0.87 (0.54, 1.40)	0.22 (0.03, 1.90)

* P-value < 0.05.

**Figure A1:**
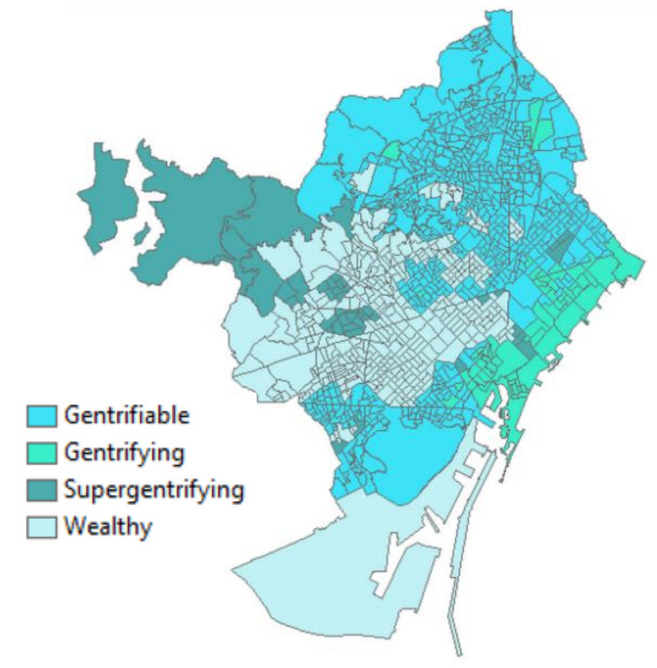
Gentrification status in Barcelona in 2006.

## Data Availability

Data are available from the authors upon request.

## Appendix A

In the BHS-2006 Mental health questionnaire—GHQ-12 (12-item Goldberg Health Questionnaire)—three dimensions are contemplated: social dysfunction (SD), anxiety and depression (DA) and loss of confidence (LC). The 12 items are the following:

1. Have you been able to concentrate on whatever you are doing? (SD)
2. Have you lost much sleep over worry? (DA)
3. Have you felt that you were playing a useful part in things? (SD)
4. Have you felt capable of making decisions about things? (SD)
5. Have you felt constantly under strain? (DA)
6. Have you felt that you could not overcome your difficulties? (DA)
7. Have you been able to enjoy your normal day-to-day activities? (SD)
8. Have you been able to face up to your problems? (SD)
9. Have you been feeling unhappy and depressed? (DA)
10. Have you been losing confidence in yourself? (LC)
11. Have you been thinking of yourself as a worthless person? (LC)
12. Have you been feeling reasonably happy all things considered? (SD)

## Appendix B

Gentrification score calculation methodology: various methods have been employed to quantitatively estimate gentrification, aiming to measure change over time across a set of demographic, real estate and new businesses or retail choices indicators at the neighborhood or census tract level [63,64]. A unified composite gentrification score is proposed in this study based on a diversity-weighted sum of social change plus change in rent values, assuming that a high amount of change across a number of social variables plus a high amount of change in median rent is the best way of defining gentrification. That is, gentrification trends are occurring only if several indicators move in the direction indicating possible gentrification [22]. For every census tract, both a social change variable and a real estate variable are measured for each point in time:

*Social change:*

- Percentage of ethnically/racially marginalized (defined as people with nationality from any of the African countries, Philippines, Peru, Pakistan, Bolivia, Ecuador, Colombia and Dominican Republic);
- Percentage of residents with university degree or higher;
- Percentage of residents with high income.

*Real estate variable:* second-hand rent prices

- Monthly rent price (€/useful m²).

The income data (1991 and 2006), the second-hand rent prices (1992 and 2006), the information on residents with high income (1991 assessed with the *Índex de Capacitat Econòmica Familiar*, ICEF, Economic Familiar Capacity Index, which represents an income value for different districts relative to the mean value of Barcelona, which adopts a reference value of 100. Thus, all neighborhoods with an ICEF > 100 are above the mean income of the city; 2006 assessed with the *Distribución Territorial de la Renta*) were obtained from the Department of Statistics of the *Ajuntament de Barcelona* [65]. The other sociodemographic data (1991 and 2006) used to calculate the gentrification score were obtained from the census and provided by IDESCAT (nationality and education level).

Z-values are calculated for all variables increases in order to assess their magnitude in relation to overall changes (positive and negative) observed throughout the study area as a whole. Such values reflect the number of standard deviations that an observation (in this case the increase in variables over a given time interval) is above or below the mean value of observations for the wider population according to the following formula:

$$Z=\frac{a-\mu }{\delta }$$

where:

*μ = mean of all scores in the study area*;

*σ = standard deviation of all scores in the study area*.

Social change variables are normalized by the Shannon Equitability score (*H_E_*), which provides a measure of both the abundance and the evenness of a group of observations:

$$H_{E}=\frac{H^{\prime }}{H_{\max}^{\prime }}=\frac{-\sum_{sv}^{m}=1p_{sv}\ln(p_{sv})}{\ln(\mathrm{>n})}$$

where:

*p_sv_* = proportion of increase in each social change variable to sum of all increases.

The final composite gentrification score for a given area and time interval. Gtract. can then be calculated using the Shannon Equitability score (*H_E_*), the n social change variable Z-values (*Z_sv_*) and the real estate Z-value (*Z_r_*) according to the following formula:

$$G_{\mathrm{tract}}{=(H}_{E}\sum_{sv=1}^{n}Z_{sv}{)+Z}_{r}$$

Once the gentrification score was obtained, it was categorized to facilitate the interpretation of the results, as other studies have done [22,36]. First, the census tracts were divided in those with a high income level in 1991 (ICEF value > 100) and those with a low income level in 1991 (ICEF value ≤ 100). Then, a second categorization was conducted by gentrification status, setting a threshold in the 90th percentile of the gentrification score (corresponding to a value of 0.864): among “low income in 1991” tracts, those with gentrification score ≤0.864 were classified as “Gentrifiable”, while those highly gentrifying (gentrification score > 0.864) were classified as “Gentrifying”. High-income areas in 1991 were considered to present a lower margin for change in the period 1991–2006, so the ones with a gentrification score ≤ p90 were considered “Wealthy”. Another category was included for wealthy areas in 1991 that still could be gentrified in the 1991–2006 period (gentrification score > p90), classified as “Supergentrifying”. Table A1 shows the distribution of census tracts by gentrification status and Figure A1 shows the graphic distribution of the gentrification status in Barcelona in 2006.

**Table A1:** Distribution of census tracts by gentrification status (absolute and relative frequencies) and Median (interquartile range, IQR) gentrification score for each category.

	N	%	Median (IQR)
**Gentrification status**			
Gentrifiable	589	55.14	0.16 (0.06–0.30)
Gentrifying	65	6.09	1.32 (1.11–1.54)
Supergentrifying	41	3.84	0.45 (0.43–0.53)
Wealthy	373	34.93	0.07 (-0.01–0.20)
Total	1068	100	0.15 (0.04–0.32)

**Table A2:** Sensitivity analyses, testing the main analyses for perceived mental health and depression/anxiety in the greenspace models without the “caregiver status” variable.

	OR	(95% CI)	P-Value		OR	(95% Ci)	P-Value		OR	(95% Ci)	P-Value
**Perceived mental health**											
Greenspace variable	All greenspace				Greenways				Parks		
without adjustment by caregiver status	0.91	(0.69, 1.21)	0.525		0.70	(0.52, 0.93)	0.016		1.07	(0.81, 1.39)	0.643
% Greenspace variable at a **100 m network** buffer	1.08	(0.82, 1.41)	0.577		0.97	(0.65, 1.45)	0.876		1.11	(0.84, 1.46)	0.461
% Greenspace variable at a **500 m network** buffer	1.00	(0.76, 1.30)	0.983		0.77	(0.58, 1.03)	0.080		0.97	(0.74, 1.27)	0.829
% Greenspace variable at a **300 m circular** buffer	1.03	(0.78, 1.35)	0.852		1.60	(0.71, 3.63)	0.258		1.09	(0.83, 1.43)	0.538
**Depression/anxiety**											
Greenspace models	All greenspace				Greenways				Parks		
without adjustment by caregiver status	0.92	(0.70, 1.20)	0.534		0.67	(0.50, 0.91)	0.009		0.96	(0.73, 1.25)	0.742
% Greenspace variable at a **100 m network** buffer	0.96	(0.73, 1.26)	0.774		0.70	(0.46, 1.07)	0.103		0.88	(0.67, 1.16)	0.371
% Greenspace variable at a **500 m network** buffer	1.11	(0.85, 1.43)	0.446		0.77	(0.58, 1.04)	0.088		1.22	(0.94, 1.58)	0.135
% Greenspace variable at a **300 m circular** buffer	1.07	(0.82, 1.40)	0.601		0.64	(0.18, 2.31)	0.492		1.10	(0.85, 1.44)	0.461

**Table A3:** Sensitivity analyses, testing main analyses for perceived mental health and depression/anxiety in the gentrification models without the “caregiver status” variable.

	OR	(95% CI)	P-Value		OR	(95% CI)	P-Value		OR	(95% CI)	P-Value
**Perceived mental health**											
*Gentrification (gentrifiable not gentrifying)*	*Gentrifying*				*Wealthy*						
Without adjustment by caregiver status	0.90	(0.56, 1.44)	0.650		0.95	(0.72, 1.26)	0.720		1.05	(0.57, 1.91)	0.885
**Depression/anxiety**											
*Gentrification (gentrifiable not gentrifying)*	*Gentrifying*				*Wealthy*						
Without adjustment by caregiver status	0.43	(0.25, 0.75)	0.003		0.80	(0.61, 1.05)	0.112		0.75	(0.38, 1.49)	0.410
